# Induction of Excess Centrosomes in Neural Progenitor Cells during the Development of Radiation-Induced Microcephaly

**DOI:** 10.1371/journal.pone.0158236

**Published:** 2016-07-01

**Authors:** Mikio Shimada, Fumio Matsuzaki, Akihiro Kato, Junya Kobayashi, Tomohiro Matsumoto, Kenshi Komatsu

**Affiliations:** 1 Department of Genome Repair Dynamics, Radiation Biology Center, Kyoto University, Kyoto, Japan; 2 Laboratory for Cell Asymmetry, Center for Developmental Biology, RIKEN, Kobe, Japan; 3 Department of Radiation System Biology, Radiation Biology Center, Kyoto University, Kyoto, Japan; University of Colorado, Boulder, UNITED STATES

## Abstract

The embryonic brain is one of the tissues most vulnerable to ionizing radiation. In this study, we showed that ionizing radiation induces apoptosis in the neural progenitors of the mouse cerebral cortex, and that the surviving progenitor cells subsequently develop a considerable amount of supernumerary centrosomes. When mouse embryos at Day 13.5 were exposed to γ-rays, brains sizes were reduced markedly in a dose-dependent manner, and these size reductions persisted until birth. Immunostaining with caspase-3 antibodies showed that apoptosis occurred in 35% and 40% of neural progenitor cells at 4 h after exposure to 1 and 2 Gy, respectively, and this was accompanied by a disruption of the apical layer in which mitotic spindles were positioned in unirradiated mice. At 24 h after 1 Gy irradiation, the apoptotic cells were completely eliminated and proliferation was restored to a level similar to that of unirradiated cells, but numerous spindles were localized outside the apical layer. Similarly, abnormal cytokinesis, which included multipolar division and centrosome clustering, was observed in 19% and 24% of the surviving neural progenitor cells at 48 h after irradiation with 1 and 2 Gy, respectively. Because these cytokinesis aberrations derived from excess centrosomes result in growth delay and mitotic catastrophe-mediated cell elimination, our findings suggest that, in addition to apoptosis at an early stage of radiation exposure, radiation-induced centrosome overduplication could contribute to the depletion of neural progenitors and thereby lead to microcephaly.

## Introduction

The International Commission on Radiological Protection (ICRP) recommends restricting the occupational radiation exposure of pregnant women because the embryo and the fetus are highly sensitive to ionizing radiation (IR) (ICRP60, 1990). For example, among the A-bomb survivors at Hiroshima and Nagasaki, microcephaly was reported in those who were exposed to radiation in utero at 9–15 weeks of gestation [1]. The incidence of microcephaly in the A-bomb survivors was approximately 50% at 1 Sv exposure, which is approximately 10 times higher than the incidence of radiation-induced tumors among the survivors. Thus, the embryonic brain is considered to be one of the tissues most vulnerable to radiation.

Radiation-induced microcephaly has been reported in rodents, including mice, which showed robust radiation-induced apoptosis mainly in progenitor cells but not neurons [2–7]. Nowak et al. showed that the DNA repair machinery processed damage more slowly in neural progenitors than in neurons [3]. Consistent with this observation, DNA-repair ability was well correlated with the induction of microcephaly [3]. Moreover, with the exception of mice that lack Artemis, which exhibit normal brain development [8], mice that are deficient in non-homologous end-joining proteins, including DNA ligase IV develop microcephaly through the unrepaired DNA double-strand breaks (DSBs) that are generated during replication [4, 9]. This difference and the mild phenotype of the Artemis-deficient mice could be explained by the finding that the cells in these mice show repair kinetics similar to that of wild-type cells at least until 6 h after irradiation [9]. However, patients with Nijmegen breakage syndrome (NBS) exhibit severe microcephaly, although they present a mild phenotype similar to that of Artemis-deficient mice [10], and the deficiency of *NBS1*, the causative gene for NBS, results in microcephaly in mice [5, 11]. Furthermore, mice lacking the breast cancer gene *Brca1* were also reported to show severe microcephaly [9, 10]. In the regulation of cellular responses, NBS1 and BRCA1 perform multiple functions, one of which is DNA repair. Therefore, a high incidence of microcephaly caused by the lack of NBS1 or BRCA1 suggests that in addition to the unrepaired-DSB-mediated apoptosis pathway, other pathways are involved in the development of microcephaly [8].

Previously, we showed that NBS1 and BRCA1 collaborate in ensuring proper centrosome duplication and that the depletion of NBS1 and BRCA1 results in the cause of excess centrosomes [5, 12–14]. Similarly, genetic disorders characterized by microcephaly, such as autosomal recessive primary microcephaly (MCPH) and ATR-Seckel syndrome, are recognized to involve defects in centrosome maintenance [15, 16]. During neurogenesis, defective spindle positioning at the apical layer is widely accepted to lead to a depletion of the progenitor pool and, consequently, to a small brain [15, 17]. However, based on studies conducted using PLK4-overexpressing mice, Marthiens et al. recently proposed the following model: the amplification of centrosomes causes a depletion of the progenitor pool by producing defects directly in cell division—rather than by impairing spindle positioning—and thus leads to microcephaly [18]. In previous studies conducted using cultured human and mouse cells, we and others showed that radiation efficiently induces centrosome overduplication, which causes defects in cell division [19, 20]. Here, we investigated whether radiation induces centrosome overduplication in the neural progenitor cells of the mouse embryonic cerebral cortex during the development of microcephaly.

## Material and Methods

### Animals and irradiation

ICR mice were purchased from SLC, Inc. (Hamamatsu, Japan), and used for all experiments. Mouse embryonic stages were calculated with the noon of the vaginal plug day serving as Embryonic Day 0.5 (E0.5). Pregnant mice were exposed to whole-body irradiation with ^137^Cs γ-rays at dose rate of 1 Gy/min delivered by a Gammacell 40 system (MDS Nordion Co., Ltd., Ottawa, Canada). After γ-ray exposure of 1 or 2 Gy, mouse embryo or brains were collected from pregnant mice and used for experiments. All mouse experiments were approved by the Institutional Animal Care and Use Committee of the RIKEN Center for Developmental Biology, and were conducted according to the guidelines for animal experiments at the RIKEN Center. On the day of the experiments, mice were euthanized through cervical dislocation and then used in the experiments. All efforts were made to minimize suffering.

### Immunofluorescence

Mouse brains were fixed in 4% paraformaldehyde in phosphate-buffered saline (PBS) at room temperature (4 h to overnight), incubated in 20% sucrose at room temperature (4 h to overnight), and then embedded in OCT compound and frozen in liquid nitrogen. Frozen brain samples were sectioned at a thickness of 14 μm (Leica cryostat CM3050) and blocked with blocking buffer (5% donkey serum in PBS), and then incubated with primary antibodies diluted in blocking buffer at room temperature (1 h to overnight). The primary antibodies were visualized using Cy3-conjugated anti-rabbit or anti-mouse IgG (Jackson ImmunoResearch, West Grove, PA) or Alexa488-conjugated anti-rat or anti-rabbit IgG (Invitrogen, Carlsbad, CA, USA. The primary antibodies used were against phospho-histone H3 (pH3) (Cell Signaling, Danvers, MA), γ-H2AX (Millipore, Billerica, MA), Sox2 (Santa Cruz Biotechnology, Santa Cruz, CA), βIII-tubulin (clone Tuj1; Covance, Princeton, NJ), ZO-1 (Invitrogen), cleaved-caspase-3 (Cell Signaling), γ-tubulin (Santa Cruz), Tbr2 (Abcam, Cambridge, UK), Brn1 (Santa Cruz), and Foxp2 (Abcam). Samples were counterstained with 4′,6-diamidino-2-phenylindole (DAPI) and analyzed using a confocal microscope (Zeiss LSM510) or a conventional microscope.

### Quantification

We quantified DAPI, cleaved-caspase-3, γ-H2AX, and pH3 staining in 200 μm wide fields with the ventricular surface positioned horizontal. All experiments were performed three times. Cells containing overduplicated centrosomes were counted as pH3-positive cells in the mouse cerebral cortex at 48 h after exposure to 1 Gy radiation.

### Statistical analysis

Statistical analysis was performed using Student’s *t* test and Excel software.

## Results

### Radiation-induced microcephaly in newborn mice

**Fig 1:**
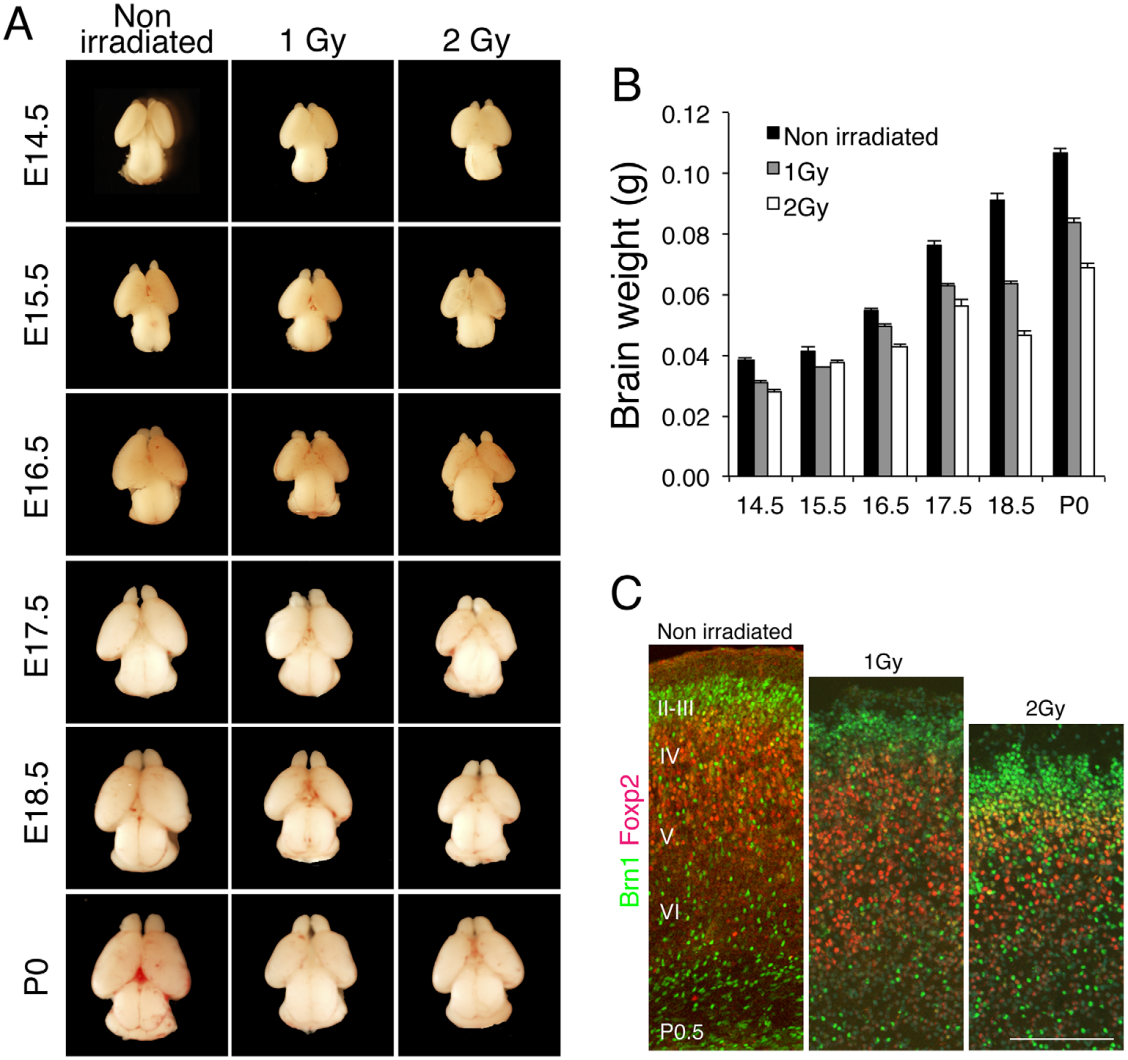
IR induces microcephaly in mice. (A) Mouse embryos at E13.5 were exposed in utero to IR of 1 or 2 Gy, and then embryonic brains were sampled at the indicated days. (B) Embryonic brains were weighed and average weights were calculated using at least 3 embryos. Error bars indicate the standard error. (C) Mouse embryos at E13.5 were exposed to 1 or 2 Gy, and then the brains of newborn mice (P0.5) were cryosectioned and stained with antibodies against Brn1 (green) and Foxp2 (red), used as markers of cerebral cortex layers II–IV and layer VI, respectively. Scale bar: 100 μm.

To investigate the effect of IR on the development of the cerebral cortex in mouse embryos, pregnant mice were exposed to 1 or 2 Gy whole-body γ-irradiation. Previous work has shown that exposure to irradiation at E13.5 in mouse, which corresponds to 9–15 weeks of gestation in human, most effectively influences the development of the cerebral cortex [1]. We irradiated E13.5 embryos in utero and measured the brain weight at the indicated day after irradiation (Fig 1A). At birth (Postnatal Day 0.5, P0.5), the average weight of the brains from embryos irradiated with 1 and 2 Gy was decreased to 78.1% and 64.1% of the weight of the unirradiated (control) newborn brain, respectively (Fig 1B). Similarly, the brain weight was decreased to 81.0% and 73.8% of control at 24 h after irradiation with 1 and 2 Gy, respectively. These results indicate that brain size was reduced after irradiation and that this reduction persisted during brain development.

Next, we measured the thickness of the cortical layers by immunostaining brain sections with antibodies against Brn1 and Foxp2 as, respectively, upper-layer (II–IV) and deep-layer (VI) markers (Fig 1C). Irradiation with 1 or 2 Gy reduced the percentage of Foxp2-positive cells to 35% and 29%, respectively, from 44% in control mice, and also markedly reduced the thickness of the deep layer in a dose-dependent manner. This result indicated that radiation exposure potently impaired mouse brain development and that this was accompanied by a thinning of the deep cortical layer.

### Radiation-induced apoptosis in neural progenitors

**Fig 2:**
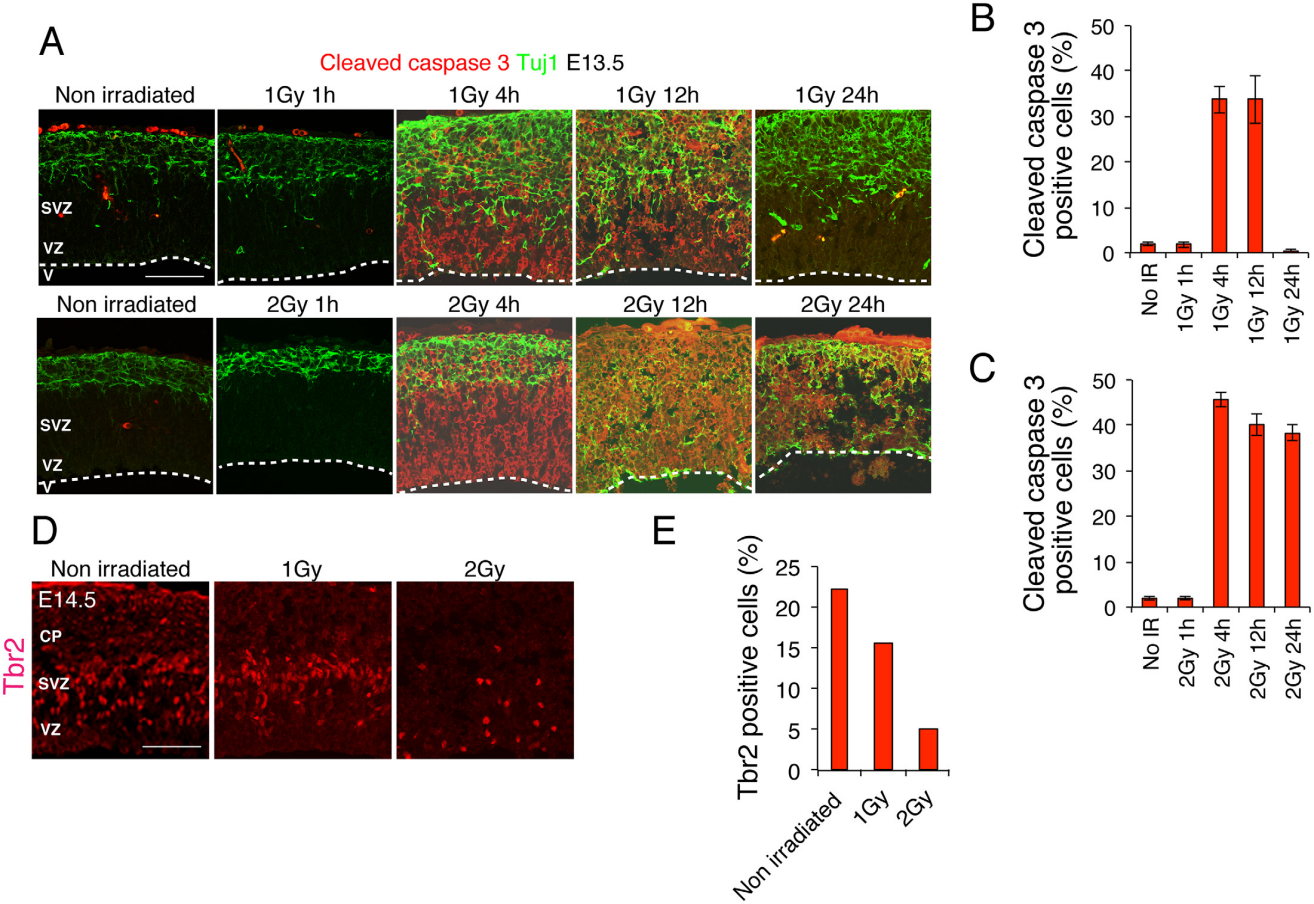
Apoptotic cell death in the mouse embryonic cerebral cortex after IR exposure. (A) Embryonic brains were sampled at 24 h after 1 or 2 Gy IR exposure and stained with antibodies against cleaved-caspase-3 (apoptosis marker) and βIII-tubulin (Tuj1; neuron marker). (B) and (C) Cleaved-caspase-3-positive cells after 1 or 2 Gy exposure were quantified. Error bars indicate the standard error. (D) Embryonic brains were sampled at 24 h after 1 or 2 Gy exposure and stained with a Tbr2 antibody (neural-progenitor marker), and then the Tbr2-positive cells were quantified. At least 3 independent mouse embryos were used for each experiment. V: ventricle; VZ: ventricular zone; SVZ: subventricular zone. The border of VZ and SVZ was delineated according to the method by Pulvers JN., et al [12]. Scale bar: 100 μm.

Because the number of Foxp2-positive cells was decreased in the irradiated newborn brain (Fig 1C), we measured the frequency of apoptotic cell death in the neural progenitors of the embryos. Embryos were irradiated in utero at E13.5 with 1 or 2 Gy, and then the brain cortical layers were immunostained at the indicated time after irradiation: we used a cleaved-caspase-3 (CCR) antibody as a marker of apoptosis and the antibody Tuj1 (βIII-tubulin) as a marker of neurons. Both 1 and 2 Gy irradiation induced apoptosis in progenitor cells but not neurons (Fig 2A), which agrees with the results reported by others [6]. After 1-Gy irradiation, 35% of the cells were apoptotic at 4 h and were eliminated 24 h later, whereas after 2-Gy irradiation, 47% of the cells were apoptotic and persisted for a comparatively longer time (Fig 2B). A similar result was obtained when apoptotic cells were detected by immunostaining the sections with a γ-H2AX antibody, even though this antibody detects cells at an early stage of apoptosis (S1 Fig). Next, to examine the status of layer-specific neural progenitors, we performed immunostaining with an antibody against Tbr2, a marker for intermediate neural progenitors, and counted the Tbr2-positive cells in the cortical layers [21]. As expected, the number of neural progenitors in the cortical layers was significantly decreased at 24 h after irradiation in a dose-dependent manner: Tbr2-positive cells, 22% in control mice, and 15% and 6% in mice irradiated with 1 and 2 Gy, respectively. The alignment of Tbr2-positive cells in the 1 Gy irradiated embryonic brain appeared similar to that in the unirradiated control, but Tbr2-positive cells had significantly reduced in the SVZ in the 2 Gy irradiated embryos.

Interestingly, when the embryonic brain was irradiated, the cerebral cortex structure was disrupted: in the irradiated mice, neurons detached from the basal layer of the cortex or the premature differentiation of neural progenitors might be induced, whereas in control mice, neurons were localized strictly within a thin layer (Fig 2A). This occurred concurrently with the appearance of apoptotic cells, and the dissociation peaked at 12 h after irradiation with 1 Gy. However, at 24 h after irradiation, in contrast to the elimination of apoptotic cells, neuronal invasion was not completely eliminated and invading neurons were still observed in the apical structure. This disrupted layer of the mouse cortex was observed comparatively more clearly in the embryos irradiated with 2 Gy. Thus, 1 Gy exposure induced considerable apoptotic cell death in neural progenitors and also a disruption of the cortical structure.

### Cell proliferation outside the apical layer

**Fig 3:**
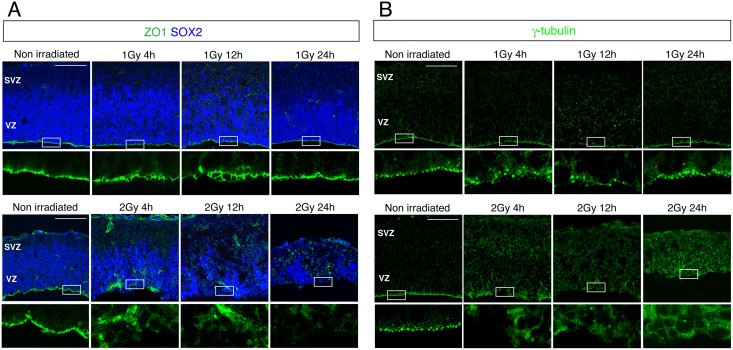
Disruption of the apical layer in the mouse embryonic cerebral cortex after IR exposure. Mouse embryos at E13.5 were irradiated with 1 or 2 Gy and the brains were sampled 24 h later and stained with antibodies against (A) ZO1 (green) and Sox2 (blue), used as markers of adherence junctions and neural progenitors, respectively; and (B) γ-tubulin (green), used as a spindle marker. VZ: ventricular zone; SVZ: subventricular zone. Scale bar: 100 μm.

Cell positioning at the apical layer is considered to play an indispensable role in the proliferation of neural progenitors [22]. Therefore, we investigated the apical structure by immunostaining with antibodies against ZO-1 and γ-tubulin, markers of adherence junctions and spindles, respectively. The ZO-1- and γ-tubulin-positive cells showed a flat bottom of the apical layer in control embryos, but this was disrupted at 4 and 24 h after 1 Gy γ-ray exposure and subsequently restored, with the peak disruption occurring at 12 h (Fig 3A). However, numerous γ-tubulin-positive cells were still detected in the basal layer, which agreed with the immunostaining observed using the Tuj1 antibody (Fig 2A). Moreover, the apical structure in the cerebral cortex was more severely disrupted in embryos irradiated with 2 Gy than in the 1 Gy irradiated embryos (Fig 3B).

**Fig 4:**
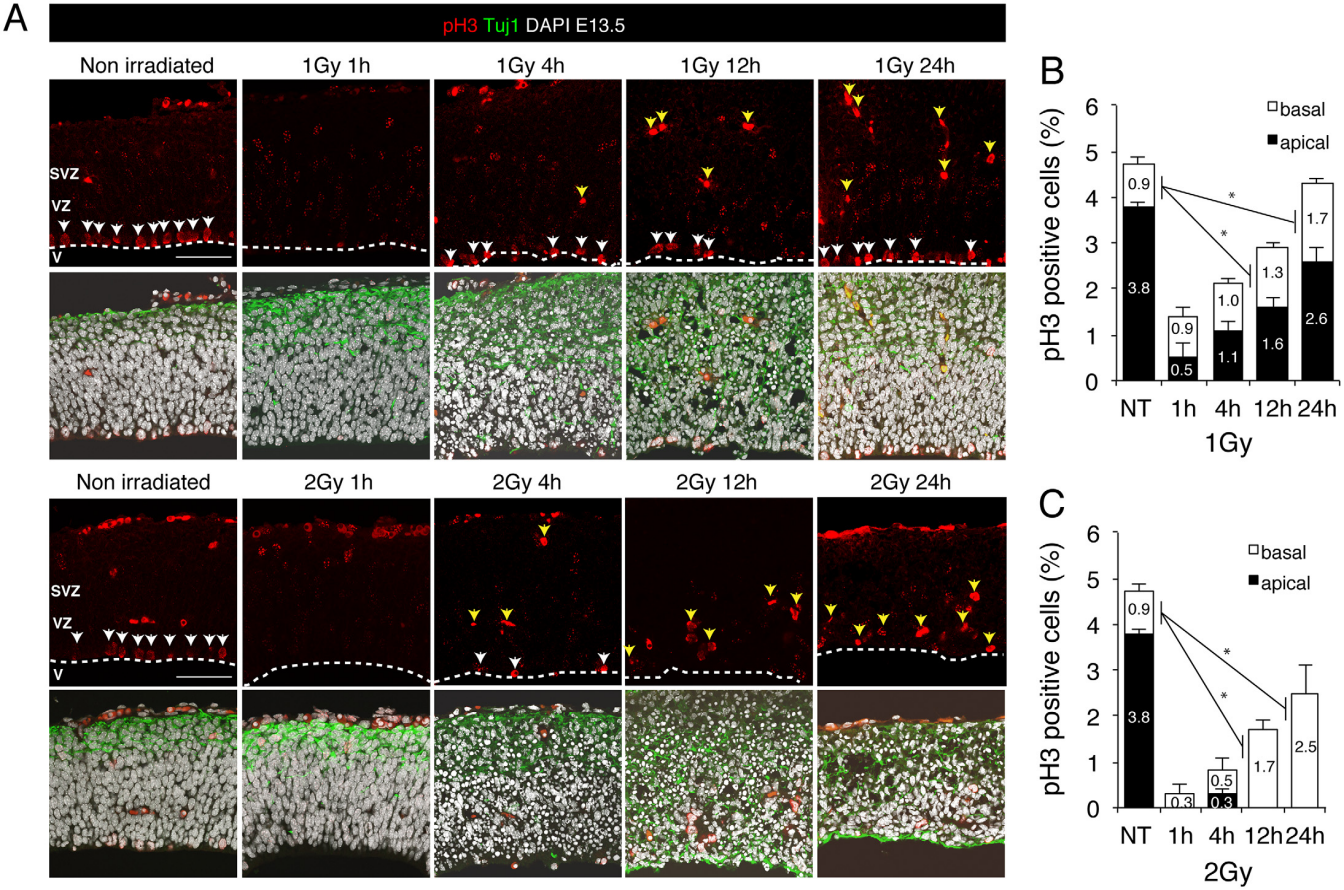
Cell proliferation in the mouse embryonic cerebral cortex after IR exposure. (A) Mouse embryos at E13.5 were irradiated with 1 or 2 Gy, and 24 h later, brains were sampled and stained with an antibody against Ser10-phosphorylated histone H3 (pH3; red, mitotic-cell marker) and the Tuj1 antibody (green, neuron marker), and counterstained with DAPI (white). Apical and basal mitotic cells are indicated by white and yellow arrows, respectively. (B) and (C) All pH3-positive cells in the apical and basal surface were quantified separately after irradiation with 1 Gy (B) or 2 Gy (C). Black and white bars indicate the apical and the basal surface, respectively. At least 3 independent mouse embryos were used for each experiment. Error bars represent the standard error. V: ventricle; VZ: ventricular zone; SVZ: subventricular zone. Scale bar: 100 μm.

To examine the proliferation of neural progenitors after irradiation, we immunostained brain sections with an antibody against histone H3 phosphorylated at Ser10, which serves as a marker of mitotic cells (Fig 4A). Mouse embryos irradiated in utero with 1 or 2 Gy showed a substantial amount of cell-cycle arrest, but cell proliferation levels were restored nearly to the control level at 24 h after 1 Gy irradiation (Fig 4A and 4B). Intriguingly, numerous γ-tubulin-positive cells and mitotic cells were observed outside the apical layer: approximately 40% of the proliferating cells at 24 h after 1 Gy irradiation (Fig 4B). These results indicated that neural progenitor cells can proliferate regardless of the spindle position at the apical layer. Our findings agree with the observation of others that a defect in spindle positioning did not impair proliferation (Figs 3 and 4) [18, 23]. Thus, although the apoptotic cells detected following 1 Gy irradiation were completely eliminated from the basal layer at 24 h after exposure, the cortical structure was not fully restored and neural progenitor cells proliferated outside the apical layer.

### Abnormal cytokinesis following IR exposure

**Fig 5:**
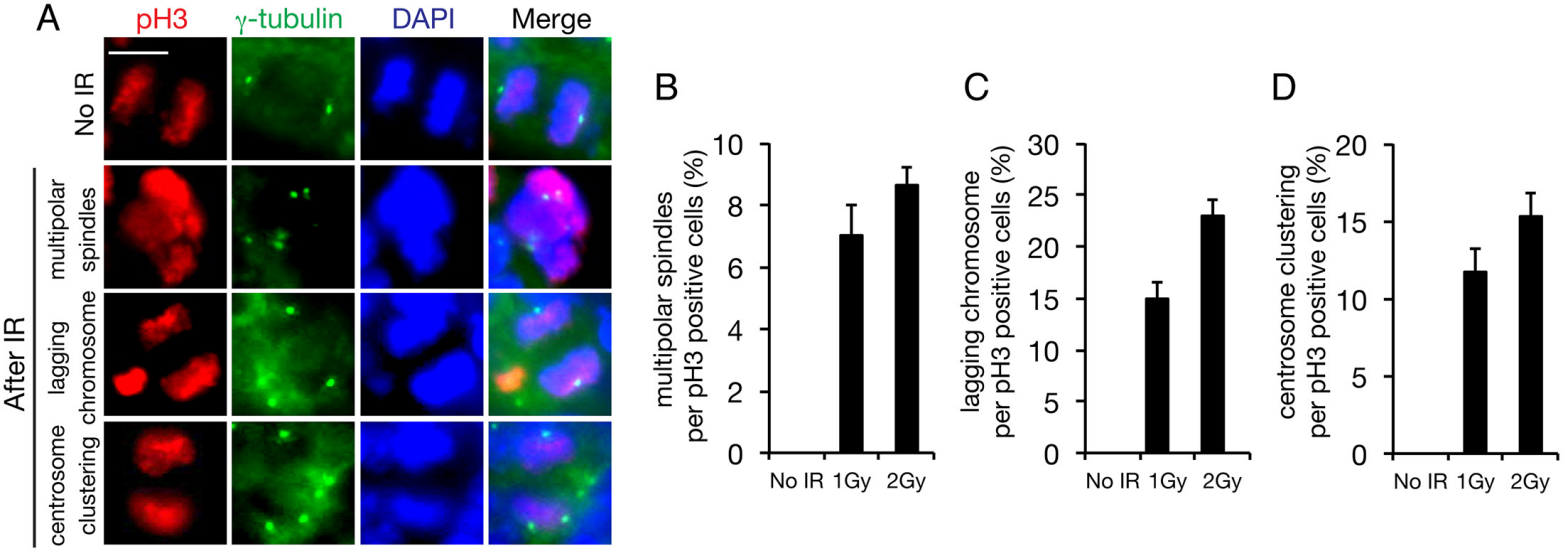
Abnormal cytokinesis in the mouse embryonic cerebral cortex after IR exposure. (A) Mouse embryos at E13.5 were irradiated with 1 or 2 Gy, and 48 h later, brains were sampled and stained with antibodies against Ser10-phosphorylated histone H3 (pH3; red in merged image) and γ-tubulin (green in merged image), used as markers of mitotic cells and centrosomes. Cells were counterstained with DAPI (blue in merged image). Quantification of (B) multipolar cell division, (C) lagging chromosomes, and (D) centrosome clustering among pH3-positive cells; at least 100 pH3-positive cells were counted. Error bars represent the standard error. Scale bar: 10 μm.

Previously, we demonstrated radiation-induced centrosome overduplication and the resulting multipolar division in cultured cells. To determine whether IR induces centrosome overduplication in the mouse cerebral cortex, we immunostained the cortical layers of embryos with a γ-tubulin antibody as a centrosome marker after IR exposure at 1 or 2 Gy. Mouse embryos were exposed to IR at E13.5 and the samples were prepared at 48 h after irradiation (Fig 5A). Whereas multipolar division occurred in 7% and 9% of the neural progenitor cells in embryos irradiated with 1 and 2 Gy, respectively, no multipolar division was detected in the progenitors in control embryos (Fig 5B). Moreover, centrosome clustering was frequently observed in 12% and 15% of progenitor cells after irradiation with 1 and 2 Gy, respectively, even if dipolar division was successful in the progenitor cells (Fig 5D). Similarly, 15% and 23% of the progenitor cells in embryos irradiated with 1 and 2 Gy, respectively, showed an induction of lagging chromosomes, which were generated as a result of centrosome clustering (Fig 5C). Multipolar division and centrosome clustering are derived from centrosome overduplication, and thus overduplication was detected in 19% and 24% of the progenitor cells after irradiation with 1 and 2 Gy, respectively. Although centrosome overduplication was detected among the cells that survived apoptosis, apoptotic cell death and abnormal cytokinesis were found to have been induced in 54% and 81% of the progenitor cells after irradiation with 1 and 2 Gy, respectively (Figs 2B, 2C, 5B and 5D). Thus, the analysis performed using apoptosis and centrosome overduplication as markers revealed that the embryonic brain is vulnerable to IR.

## Discussion

We have shown here for the first time that radiation induces centrosome overduplication in neural progenitor cells during the development of microcephaly. When embryos at E13.5 were exposed to 1 or 2 Gy, the irradiation induced microcephaly, in which the thickness of the brain cortical layer was reduced markedly in a dose-dependent manner (Fig 1). Growth arrest occurred for a period following 1 Gy irradiation but proliferation levels were restored to the control level after 24 h, and the effect observed was comparatively more severe when 2 Gy irradiation was used (Fig 4). Among these proliferating cells, aberrant cytokinesis was induced in 19% and 34% of the progenitors at 48 h after irradiation with 1 and 2 Gy, respectively (Fig 5). In the case of 1 Gy irradiation, the abnormal cytokinesis included 7% multipolar division and 12% centrosome clustering, which could lead to a depletion of the progenitor pool through cell death and to brain degeneration through lagging chromosomes, respectively [18, 24, 25].

As reported previously by others [2–7], we observed a significant amount of IR-triggered apoptotic cell death, in 35% and 40% of neural progenitors at 4 h after irradiation with 1 and 2 Gy, respectively (Fig 2). Consequently, apoptosis induced a depletion of the neural progenitor cells 24 h later: progenitor cells, 15% in 1 Gy irradiated mice versus 23% in control mice. Although the apoptotic cells were completely eliminated at 24 h after 1 Gy irradiation, a considerable amount of abnormal cytokinesis was induced 48 h later. We previously showed that centrosome overduplication occurred in cultured U2OS cells at 48 h after irradiation and that it peaked at 96 h post-irradiation [19]. Each neural progenitor cell is known to divide into 2 daughter progenitors through symmetric division during a limited period, which starts at E8 and ends at E16.5 [26]. Therefore, present results proposed an alternative pathway of radiation-induced microcephaly that, in addition to the apoptosis at E13.5–E14.5 as reported previously, subsequent centrosome overduplication at E15–E17.5 profoundly affect neurogenesis by depleting the progenitor pool.

The frequency of radiation-induced centrosome overduplication during neurogenesis that was measured here is considerably higher than that we previously measured in U2OS cells: 19% in neural progenitors after 1 Gy irradiation (Fig 5) versus 3.5% in U2OS cells after 5 Gy irradiation [19]. Although the mechanism underlying high-frequency centrosome overduplication in neural progenitors remains to be elucidated, one potential explanation is an association of centrosome induction with the expression level of p21, an inhibitor of CDK2-CyclinA/E: High p21 expression can reduce centrosome overduplication, because centrosomes are overduplicated as a result of CDK2-CyclinA/E activation [27]. Notably, we showed previously that genistein, an isoflavonoid, reduced radiation-induced centrosome overduplication by upregulating p21 [28]. Conversely, p21 expression in neural progenitor cells was reported to be lowered markedly following IR exposure [6]. This insufficient p21 expression might lead to excessive centrosome overduplication in neural progenitors, although it would also concurrently abolish the p21-mediated G1/S checkpoint in the presence of DNA damage. Inactivation of the G1/S checkpoint has been suggested to potentially prevent the differentiation of neural progenitors into neurons and thus favor the reconstitution of the neural progenitor pool, because lengthening of G1 by the G1/S checkpoint promotes this differentiation [29]. Consequently, insufficient p21 expression in irradiated neural progenitor cells could contribute to enhanced centrosome overduplication, which leads to microcephaly through the depletion of progenitor cells.

As noted in the introduction section, a previous study showed that centrosome overduplication associated with PLK4 overexpression caused microcephaly by affecting cell division (i.e., by triggering multipolar cell division) [18]. Moreover, in mice, loss-of-function mutations in *CDK5RAP2*, the causative gene for the inherited disease MCPH3, resulted in the microcephaly phenotype, and CDK5RAP2-mutant mouse embryonic fibroblasts showed centrosome overduplication [30]. These findings suggest that centrosome overduplication could be associated with improper brain development.

Centrosome overduplication has been reported to be associated with aneuploidy, which is a tumor hallmark. Therefore, centrosome overduplication might be involved in brain tumor development. Although neural progenitor cells divide most actively in the embryonic brain, their cell division continues in the adult brain. Further investigation is required to clarify the adverse effects of centrosome overduplication during neurogenesis in the embryo and tumorigenesis in the adult brain after medical use of radiation, including radiation therapy and radiosurgery.

## Supporting Information

S1 Fig DNA damage in the mouse embryonic cerebral cortex after IR exposure. (A) Embryonic brains were sampled at 24 h after IR exposure at 1 or 2 Gy and stained with a γ-H2AX antibody, which was used as a marker of radiation-induced DNA damage and apoptosis. These effects were distinguished according to the shape of γ-H2AX staining: dot-like staining, DNA damage; pan-staining, apoptosis. (B) and (C) Quantification of DNA damage (orange) and apoptosis (red).
(PDF)
