# Supplementary figures and images for: Induction of Excess Centrosomes in Neural Progenitor Cells during the Development of Radiation-Induced Microcephaly
Source: PLoS One. 2016 Jul 1;11(7):e0158236. doi: 10.1371/journal.pone.0158236 (PMC4930206; doi:10.1371/journal.pone.0158236)

Supplementary figure. 1

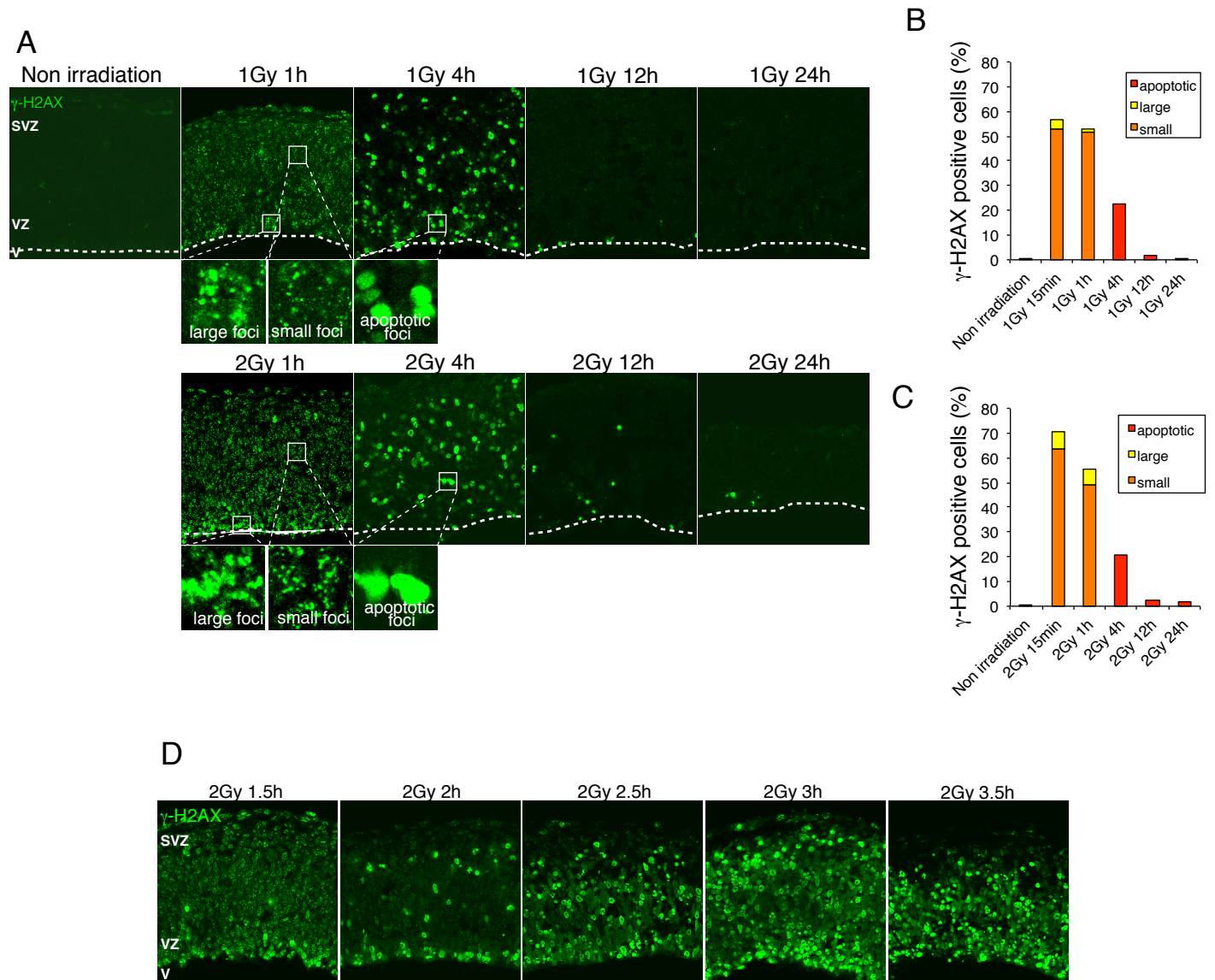

Supplement: S1 Fig — (A) Embryonic brains were sampled at 24 h after IR exposure at 1 or 2 Gy and stained with a γ-H2AX antibody, which was used as a marker of radiation-induced DNA damage and apoptosis. These effects were distinguished according to the shape of γ-H2AX staining: dot-like staining, DNA damage; pan-staining, apoptosis. (B) and (C) Quantification of DNA damage (orange) and apoptosis (red). (PDF) [file pone.0158236.s001.pdf]
